# Real‐World Study on the Impact of Novel Coronavirus Infection on Male Erectile Function

**DOI:** 10.1002/ccr3.71455

**Published:** 2025-11-23

**Authors:** Chuhong Chen, Zihan Zhou, Zhecheng Zhang, Qingtong Yi, Rujian Zhu, Xujun Xuan, Dong Liu

**Affiliations:** ^1^ State Key Laboratory of Reproductive Medicine and Offspring Health, Center for Reproductive Medicine, Institute of Women, Children and Reproductive Health Shandong University Shandong China; ^2^ Department of Urology, Shanghai Pudong Hospital Fudan University Pudong Medical Center Shanghai China; ^3^ Hebei Medical University Shijiazhuang China; ^4^ Department of Andrology, Seventh Affiliated Hospital Sun Yat‐sen University Shenzhen China

**Keywords:** COVID‐19, male erectile dysfunction, novel coronavirus infection, sexual function, survey and questionnaire

## Abstract

This study aims to examine the impact of novel coronavirus (COVID‐19) infection on male erectile function and to identify potential influencing factors. Male participants aged 18–65 years from Beijing and Shanghai were included in the study. A post‐COVID‐19 sexual function questionnaire was utilized for data collection. The SPSS 23.0 software facilitated data analysis, employing mean ± standard deviation to describe continuous variables. A total of 1098 valid questionnaires were collected. The findings indicate a significant increase in sexual drive across all age groups following COVID‐19 infection, with notable erection difficulties in the young and middle‐aged groups. Compared to uninfected individuals, infected men reported a significant decline in total sexual function scores and overall satisfaction, with markedly lower satisfaction noted in the youth group. Post‐COVID‐19 infection, there is an increase in male sex drive across all age groups. Erectile difficulties notably escalated in the young and middle‐aged groups, and there was a general decrease in the total scores of sexual function and satisfaction, particularly among the young, whereas the middle‐aged and elderly groups were less impacted.

**Abbreviations**
COVID‐19: novel coronavirus
ED: erectile dysfunction
IIEF‐15: International Index of Erectile Function
PDE5i: 5 phosphodiesterase inhibitor
PHEIC: public health emergency of international concern
ROS: reactive oxygen species

**Summary**

- Our real‐world study of 1098 Chinese males revealed:
  ○ COVID‐19 significantly increases erectile dysfunction risk, especially in young (18–29) and middle‐aged men (30–44).
  ○ Sexual function and satisfaction decline post‐infection, most pronounced in youth.
  ○ COVID‐19 is a major ED risk factor, advocating for targeted interventions in younger populations and further mechanistic research.

## 1 Introduction

On May 5, 2023, Tedros Adhanom Ghebreyesus, the Director‐General of the World Health Organization, declared that COVID‐19 was no longer considered a public health emergency of international concern (PHEIC). In China, as of December 26, 2022, it was announced that COVID‐19 would transition from a Class A to a Class B infectious disease effective January 8, 2023, reflecting a significant shift in epidemic response policies. Under the revised management protocols, neither infected individuals nor their close contacts are required to quarantine, which led to an initial surge in infections. Understanding the potential long‐term effects of COVID‐19 infection is crucial. Clinical studies have demonstrated an increase in the incidence of erectile dysfunction following COVID‐19 infection [1]. By employing a questionnaire survey and statistical analysis of multiple indicators of male sexual function before and after COVID‐19 infection, this study presents statistical conclusions and explores potential mechanisms to elucidate the effects of COVID‐19 on changes in male erectile function. (Pre‐infection status: For the COVID‐19 cohort, pre‐infection sexual function was assessed retrospectively using validated questionnaires (e.g., International Index of Erectile Function [IIEF‐15]) during patient interviews. Participants were asked to recall their sexual function in the 3–6 months preceding their COVID‐19 diagnosis. Post‐infection status: Sexual function was re‐evaluated at 3 and 6 months after COVID‐19 resolution (confirmed via negative PCR).)

## 2 Objective and Methods

### 2.1 Research Object

The study included male participants from Shanghai and Beijing, aged between 18 and 65 years, all with prior sexual experience. The questionnaire was collected from May 2023 to the end of July 2023, covering the post‐infection population and uninfected population of COVID‐19. The target was to recruit 1500 respondents.

### 2.2 Materials and Methods

#### 2.2.1 Questionnaire Survey Was Adopted in This Study

The IIEF‐15 was utilized to assess the erectile function of participants over the past 4 weeks. The IIEF‐15 comprises 15 questions addressing five domains of sexual function: Erectile Function (EF), Orgasmic Function (OF), Sexual Desire (SD), Intercourse Satisfaction (IS), and Overall Satisfaction (OS) [2].

The IIEF‐15 Questionnaire utilizes a detailed scoring method where questions 1–10 are scored on a 6‐point scale ranging from 0 to 5, and questions 11–15 on a 5‐point scale from 1 to 5. The grading system aggregates scores for specific sections: questions 1–5 and 15 determine the IIEF‐EF score, categorizing erectile function as follows: 26–30 points indicate no erectile dysfunction, 17–25 points suggest mild dysfunction, 11–16 points denote moderate dysfunction, and 10 or fewer points signify severe dysfunction. Additionally, the sum of scores for questions 6–8 yields the sexual satisfaction score, questions 9 and 10 provide the orgasmic function score, questions 11 and 12 assess sexual desire, and questions 13 and 14 measure overall satisfaction.

A custom‐designed questionnaire was also employed to gather essential data on participants, including age, marital status, height, weight, occupation, monthly income, lifestyle and behavioral habits, residence, vaccination status, and COVID‐19 infection status. This information was rated using a five‐point Likert scale, with higher scores indicating better sexual function.

#### 2.2.2 Inclusion and Exclusion Criteria

##### 2.2.2.1 Inclusion Criteria

(1) Male over 18 years old; (2) having a spouse or permanent sexual partner; (3) have lived locally for more than 1 year; (4) understand the content of the questionnaire and can cooperate with the investigation.

##### 2.2.2.2 Exclusion Criteria

(1) Non‐sexual experience; (2) not cooperating with investigators; (3) a history of chronic disease; (4) there are bad addicts such as tobacco and alcohol; (5) people with erectile dysfunction before COVID‐19 infection; (6) people taking sexual function‐enhancing medications within 6 months prior to assessment and before it ended.

The study protocol was approved by the Ethics Committee of Pudong Hospital affiliated with Fudan University, adhering to the principle of voluntary participation, and informed consent to participate was obtained from all of the participants in the study. Researchers provided detailed information about the study to participants before conducting the survey. Participants completed the questionnaire anonymously using the mobile application, Questionnaire Star, ensuring privacy protection.

### 2.3 Questionnaire Survey Method

The questionnaire survey method (Supporting Information S1) employed in this study involved the distribution of questionnaires by investigators who were uniformly trained. Initially, the investigators provided uniform guidance to explain the purpose, significance, and method of filling out the questionnaire to the subjects. After obtaining their consent, the questionnaires were distributed and completed by the subjects on the spot. A total of 1500 questionnaires were planned for distribution.

### 2.4 Statistical Methods

Statistical analysis was performed using SPSS 23.0 software. Continuous variables were described using mean ± standard deviation, while categorical variables were represented as a number (percentage). Basic demographic differences among study participants were analyzed using *t*‐tests, Chi‐square tests, or Fisher's exact tests. Differences in erectile function between COVID‐19 infected and uninfected individuals were assessed using Chi‐square or Fisher's exact tests. Following the identification of covariates through univariate analysis, a logistic regression model was developed to explore the association between COVID‐19 infection and male erectile dysfunction. Differences in male sexual function before and after COVID‐19 infection were compared using paired sample *t*‐tests.

## 3 Results

### 3.1 Questionnaire Collection Status

In this survey, 1500 questionnaires were distributed, and 1098 were returned with valid responses, including 905 from Beijing and 193 from Shanghai. The basic demographic information of the participants is detailed in Table 1.

**TABLE 1:** Basic information of the research object (*n* = 1098).

	Total	Beijing	Shanghai
	*n* = 1098	*n* = 905	*n* = 193
Age	31.2 ± 5.6	30.9 ± 4.8	32.9 ± 8.0
Marriage			
Unmarried	430 (39.1)	301 (33.3)	129 (66.8)
Married	666 (60.7)	601 (66.7)	62 (32.2)
Divorced	2 (0.2)	0 (0.0)	2 (1.0)
Height			
< 165 cm	589 (53.6)	582 (64.3)	7 (3.6)
165–174.9 cm	385 (35.1)	298 (32.9)	87 (45.1)
175–184.9 cm	110 (10.0)	19 (2.1)	91 (47.2)
≥ 185 cm	14 (1.3)	6 (0.7)	8 (4.1)
Weight		62.5 ± 8.1	75.6 ± 19.4
Occupation			
White collar	404 (36.8)	276 (30.5)	128 (66.3)
Blue collar	602 (54.8)	569 (62.9)	33 (17.1)
Other	92 (8.4)	60 (6.6)	32 (16.6)
Education			
Junior high school and below	76 (6.9)	73 (8.1)	3 (1.6)
Senior high school	186 (16.9)	174 (19.2)	12 (6.2)
College	457 (41.6)	362 (40.0)	95 (49.2)
Graduate and above	379 (34.6)	296 (32.7)	83 (43.0)
Monthly income			
< 2000 Yuan	18 (1.6)	14 (1.5)	4 (2.1)
2000–3999 Yuan	459 (41.8)	455 (50.3)	4 (2.1)
4000–6999 Yuan	4445 (40.5)	428 (47.3)	17 (8.8)
> 7000 Yuan	176 (16.0)	8 (0.9)	168 (87.0)
Smoking			
Never	223 (20.3)	90 (9.9)	133 (68.9)
Sometimes	327 (29.8)	312 (34.5)	15 (7.8)
Usually	312 (28.4)	294 (32.5)	18 (9.3)
Quit smoking	236 (21.5)	209 (23.1)	27 (14.0)
Alcohol			
Never	44 (4.0)	4 (0.4)	40 (20.7)
Seldom	454 (41.3)	346 (38.2)	454 (41.3)
Sometimes	372 (33.9)	336 (37.1)	36 (16.7)
Usually	223 (20.3)	218 (24.1)	5 (2.6)
Often	5 (0.5)	1 (0.1)	4 (2.1)
Exercise			
Never	88 (8.0)	71 (7.8)	17 (8.8)
Seldom	263 (24.0)	210 (23.2)	53 (27.5)
Sometimes	402 (36.6)	323 (35.7)	79 (40.9)
Usually	345 (31.4)	301 (33.3)	44 (22.8)
COVID‐19 infection			
Uninfected	108 (9.8)	83 (9.2)	25 (13.0)
Infected	990 (90.2)	822 (90.8)	168 (87.0)
Get vaccinated against COVID‐19			
Never vaccinated	11 (1.0)	1 (0.1)	10 (5.2)
Get one dose	159 (14.5)	143 (15.8)	16 (8.3)
Get two dose	549 (50.0)	299 (33.0)	80 (41.5)
Get three dose or more	379 (34.5)	462 (51.0)	87 (45.1)

### 3.2 Changes in Erectile Function Before and After COVID‐19 Infection

Table 2 presents the fundamental details of both the infected and uninfected individuals in the study (*p* > 0.05).

**TABLE 2:** The fundamental details of the infected and uninfected individuals in the study (*n* = 1098).

	Total	Uninfected	Infected	*p*
	*n* = 1098	*n* = 990	*n* = 108	
Age, years	31.3 ± 5.6	31.1 ± 5.4	32.6 ± 6.5	0.054
Height				0.201
< 165 cm	589 (53.6)	541 (54.6)	48 (44.4)	
165–174.9 cm	385 (35.1)	342 (34.5)	43 (35.1)	
175–184.9 cm	110 (10.0)	95 (9.6)	15 (13.9)	
≥ 185 cm	14 (1.3)	12 (1.2)	2 (1.9)	
Weight, kg	64.7 ± 12.0	64.5 ± 11.4	66.3 ± 16.1	0.299
Occupation				0.152
White collar	404 (36.8)	363 (36.7)	41 (38.0)	
Blue collar	602 (54.8)	549 (55.5)	53 (54.8)	
Other	92 (8.4)	78 (7.9)	14 (13.0)	
Monthly income				0.212
< 2000 Yuan	18 (1.6)	16 (1.6)	2 (1.9)	
2000–3999 Yuan	459 (41.8)	154 (15.6)	22 (20.4)	
4000–6999 Yuan	4445 (40.5)	409 (41.3)	50 (46.3)	
> 7000 Yuan	176 (16.0)	411 (41.5)	34 (31.5)	
Smoking				0.914
Never	223 (20.3)	201 (20.3)	22 (20.4)	
Sometimes	327 (29.8)	296 (29.9)	31 (28.7)	
Usually	312 (28.4)	283 (28.6)	29 (26.9)	
Quit smoking	236 (21.5)	210 (21.2)	26 (24.1)	
Alcohol				0.546
Never	44 (4.0)	37 (3.7)	7 (6.5)	
Seldom	454 (41.3)	412 (41.6)	42 (38.9)	
Sometimes	372 (33.9)	339 (34.2)	33 (30.6)	
Usually	223 (20.3)	198 (20.0)	25 (23.1)	
Often	5 (0.5)	4 (0.4)	1 (0.9)	
Exercise				0.127
Never	88 (8.0)	73 (7.4)	15 (13.9)	
Seldom	263 (24.0)	235 (23.7)	28 (25.9)	
Sometimes	402 (36.6)	368 (37.2)	34 (31.5)	
Usually	345 (31.4)	314 (31.7)	31 (28.7)	
Region				0.109
Beijing	905 (82.4)	822 (83.0)	83 (76.9)	
Shanghai	193 (17.6)	168 (17.0)	25 (23.1)	

*Note:* The student *t*‐test was utilized for continuous variables, while the Pearson Chi‐square test or Fisher exact test was employed for categorical variables. There were no significant differences in basic information between the infected and uninfected groups (*p* > 0.05).

The analysis of changes in erectile function before and after COVID‐19 infection is presented in Table 3. It highlights significant differences in the total scores of sexual function (*p* = 0.031) and overall satisfaction (*p* < 0.001) between infected and uninfected individuals, with a higher proportion of normal scores observed among the uninfected. However, no significant differences were found in erectile function (*p* = 0.056), orgasm (*p* = 0.085), or sexual desire (*p* = 0.616).

**TABLE 3:** Erectile function in infected and uninfected persons with COVID‐19 (*n* = 1098).

	Total	Infected	Uninfected	*p*
	*n* = 1098	*n* = 990	*n* = 108	
Total score of sexual function				
Normal > 60	158 (14.4)	135 (13.6)	23 (21.3)	0.031*
Abnormal ≤ 60	940 (85.6)	855 (86.4)	85 (78.7)	
Erectile function				
Normal > 25	174 (15.8)	150 (15.2)	24 (22.2)	0.056***
Abnormal ≤ 25	924 (84.2)	840 (84.8)	84 (77.8)	
Orgasmic function				
Normal > 8	198 (18.0)	172 (17.4)	26 (24.1)	0.085***
Abnormal ≤ 8	900 (82.0)	818 (82.6)	82 (75.9)	
Sexual desire				
Normal > 8	88 (8.0)	78 (7.9)	10 (9.3)	0.616***
Abnormal ≤ 8	1010 (92.0)	912 (92.1)	98 (90.7)	
Intercourse satisfaction				
Normal > 12	121 (11.0)	112 (11.3)	9 (8.3)	0.348***
Abnormal ≤ 12	977 (89.0)	878 (88.7)	99 (91.7)	
Overall satisfaction				
Normal > 12	128 (11.7)	104 (10.5)	24 (22.2)	< 0.001**
Abnormal ≤ 12	970 (88.3)	886 (89.5)	84 (77.8)	

*Note:* There were significant differences in the total score of sexual function between infected and uninfected persons (*p < 0.05). There is an extremely significant difference in overall satisfaction (**p < 0.001). There were no significant differences in erectile function, orgasm, sexual desire and sexual satisfaction between infected and uninfected persons (***p > 0.05).

Table 4 presents the results of a logistic regression analysis exploring the impact of COVID‐19 infection on male erectile function. The analysis reveals that the risk of experiencing declines in total sexual function scores (*p* = 0.04) and overall satisfaction (*p* = 0.01) is 2.62 and 2.30 times higher, respectively, in infected individuals compared to those uninfected. These findings are consistent with those reported in Table 3, demonstrating a decrease in both total sexual function scores and overall satisfaction following COVID‐19 infection.

**TABLE 4:** Logistic regression analysis of COVID‐19 infection and male erectile function (*n* = 1098).

	Total score of sexual function		Erectile function		Orgasmic function		Sexual desire		Intercourse satisfaction		Overall satisfaction	
	OR (95% CI)	*p*	OR (95% CI)	*p*	OR (95% CI)	*p*	OR (95% CI)	*p*	OR (95% CI)	*p*	OR (95% CI)	*p*
Uninfected	Ref.		Ref.		Ref.		Ref.		Ref.		Ref.	
Infected	2.62 (1.05–6.57)*	0.04*	2.38 (0.91–6.19)	0.08	1.51 (0.75–3.00)	0.25	0.96 (0.45–2.02)	0.91	0.67 (0.30–1.50)	0.32	2.30 (1.27–4.17)*	0.01 **

*Note:* The total score of sexual function in the infected group was significantly different from that in the uninfected group (*p < 0.05). There was a significant difference in overall satisfaction (**p ≤ 0.01).

### 3.3 Comparative Analysis of Male Sexual Function Before and After COVID‐19 Infection

Table 5 presents data on sexual function across different age groups, showing the following results: (1) The frequency of sexual impulses significantly increased across all age groups post‐infection (*p* < 0.001). (2) There was an increase in the difficulty of achieving an erection after infection, particularly noticeable in the young and middle‐aged groups (*p* = 0.014). (3) Satisfaction with sexual life significantly declined in the youth group (*p* = 0.036). (4) The scores for the middle‐aged group were higher than those of the young and combined young and middle‐aged groups, possibly attributed to the smaller sample size (*n* = 16). This table highlights age‐related variations in the impact of COVID‐19 on sexual function.

**TABLE 5:** Comparison of male sexual function before and after COVID‐19 infection (*n* = 990).

	Total			Youth group			Young to middle‐aged group			Middle to old age group		
				*n* = 381			*n* = 593			*n* = 16		
	Before infection	After infection	*p*	Before infection	After infection	*p*	Before infection	After infection	*p*	Before infection	After infection	*p*
Sex impulse frequency	1.60 ± 1.08	1.76 ± 1.17	< 0.001**	1.67 ± 1.14	1.82 ± 1.20	0.002*	1.52 ± 1.03	1.70 ± 1.15	< 0.001**	2.63 ± 0.72	2.50 ± 0.73	0.164***
Sex impulse level	1.69 ± 1.17	1.73 ± 1.17	0.355	1.84 ± 1.17	1.78 ± 1.19	0.312	1.59 ± 1.17	1.63 ± 1.17	0.063	2.06 ± 0.78	2.13 ± 0.72	0.580
Erection after sexual stimulation	1.80 ± 1.25	1.75 ± 1.25	0.294	1.86 ± 1.31	1.85 ± 1.31	0.788	1.73 ± 1.21	1.68 ± 1.20	0.311	2.75 ± 0.58	2.50 ± 0.97	0.104
Firmly complete sexual intercourse	1.83 ± 1.28	1.83 ± 1.29	0.937	1.95 ± 1.31	1.94 ± 1.31	0.894	1.74 ± 1.25	1.74 ± 1.26	0.947	2.69 ± 0.95	2.56 ± 0.96	0.333
Erection difficulty	1.95 ± 1.35	1.87 ± 1.32	0.025*	2.03 ± 1.39	1.99 ± 1.39	0.480	1.86 ± 1.31	1.75 ± 1.27	0.014*	3.06 ± 1.18	3.31 ± 0.79	0.388
Ejaculation difficulty	1.90 ± 1.37	1.88 ± 1.35	0.643	1.98 ± 1.38	1.99 ± 1.39	0.808	1.81 ± 1.34	1.77 ± 1.31	0.402	3.25 ± 1.18	3.31 ± 0.87	0.718
Decreased sex drive is the problem	2.80 ± 1.29	2.82 ± 1.30	0.566	2.87 ± 1.38	2.95 ± 1.36	0.200	2.74 ± 1.23	2.72 ± 1.26	0.648	3.50 ± 0.97	3.75 ± 1.07	0.164
Induce or maintain an erection is a problem	2.85 ± 1.31	2.85 ± 1.31	0.979	2.94 ± 1.38	2.94 ± 1.37	0.966	2.76 ± 1.25	2.77 ± 1.27	0.808	4.06 ± 1.07	3.75 ± 1.24	0.173
Ejaculation is a problem	2.89 ± 1.34	2.87 ± 1.32	0.548	3.01 ± 1.36	2.95 ± 1.36	0.333	2.78 ± 1.32	2.79 ± 1.29	0.861	4.19 ± 1.11	3.88 ± 1.09	0.136
Sexual satisfaction	2.60 ± 1.68	2.49 ± 1.71	0.062	2.72 ± 1.72	2.52 ± 1.74	0.036*	2.50 ± 1.63	2.45 ± 1.67	0.505	3.63 ± 1.86	3.38 ± 2.06	0.362

*Note:* Youth group: 18–29 years old; Young to middle‐aged group: 30–44 years old; Middle to old age group: Age 45 and older. The frequency of total sexual impulse increased after COVID‐19 infection compared with before COVID‐19 infection (**p < 0.01), and this is especially evident in the youth group and the young to middle‐aged group (**p < 0.01), and there was no significant difference between the middle to old age groups (***p > 0.05). Overall erection difficulty increased (*p < 0.05), and the young and middle‐aged group was the most significant. Satisfaction with sex was significantly lower in the youth group (*p < 0.05).

## 4 Discussion

As national epidemic prevention policies become more relaxed, an increase in COVID‐19 infections is inevitable. Early in the pandemic, some media outlets reported that the virus might impair male sexual function and fertility, causing concern among many men, particularly those infected. Previous studies have indicated that individuals with COVID‐19 are at an elevated risk of experiencing erectile difficulties [3].

Although a significant number of COVID‐19 patients report challenges with erectile function during their infection period, scientific evidence directly linking COVID‐19 to erectile dysfunction remains unconfirmed. This gap in knowledge forms the basis of our study, which aims to explore the real‐world impact of COVID‐19 on male erectile function. The findings of this study are as follows:

Firstly, there has been a notable increase in the use of sexual function drugs during the COVID‐19 pandemic. Common drugs such as sildenafil citrate, a type 5 phosphodiesterase inhibitor (PDE5i), are believed to improve erectile dysfunction (ED). This surge may be attributed to symptoms like weakness and fatigue caused by COVID‐19, adversely affecting sexual function. Psychological stresses such as anxiety and depression, also prevalent during infections, likely contribute to this trend. Consequently, some individuals might seek sexual function drugs to enhance their quality of life.

Secondly, the frequency of sexual impulses has increased across all age groups post‐infection. This change could be driven by increased levels of anxiety and depression, along with diminished sleep quality during the recovery phase of COVID‐19 [4]. Engaging in sexual activity may alleviate stress, primarily through the release of “pleasure chemicals” like oxytocin, beta‐endorphins, and dopamine [5]. Additionally, COVID‐19 may disrupt the endocrine system, altering hormone secretion, such as testosterone, which in turn affects sexual impulses [6].

Despite the findings of this study, previous research has indicated that individuals with COVID‐19 infections lasting ≥ 12 weeks experience a decreased frequency of sexual impulse [7]. This discrepancy might be attributed to subjective interpretations of sex drive levels.

Furthermore, post‐infection erectile difficulty has notably increased, especially among young and middle‐aged groups. Potential mechanisms for this increase in erectile difficulty include: (1) The novel coronavirus may damage male reproductive structures such as the testicles, epididymis, and seminal vesicles through its known receptor ACE2, directly impacting male sexual function [8, 9]. (2) The virus can cause pulmonary tissue fibrosis, leading to dyspnea, hypoxemia, and other symptoms [10, 11, 12, 13], which reduce blood oxygen saturation and affect nitric oxide (NO) production. This impacts blood vessel dilation, contributing to organic erectile dysfunction. (3) COVID‐19 infection results in down‐regulation of ACE2 and disrupted transformation of angiotensin II (Ang II), increasing Ang II levels and reducing angiotensin 1–7 (Ang1–7) production. Ang1–7 has anti‐inflammatory and antioxidant properties [14], and its reduction may enhance reactive oxygen species (ROS) production, inducing oxidative stress and inflammatory cytokine accumulation, thereby causing endothelial dysfunction and erectile dysfunction.

A study by Roman scholar Emmanuele A. Jannini, which involved 100 men (25 CoV‐positive and 75 CoV‐negative), found that the risk of ED in men infected with SARS‐CoV‐2 was over five times higher than in those uninfected [15]. This supports our results.

The impact is particularly significant in the young and middle‐aged groups, likely because (1) young and middle‐aged individuals generally have better health and fewer comorbidities, making the physical changes more noticeable and the development of ED more pronounced in this demographic. (2) The psychological stress from COVID‐19 infection, such as anxiety, fear, and depression, may have a more significant impact on the sexual function of young and middle‐aged people, whereas older adults might better adapt to these stresses.

Finally, the study identified a decline in both the total score of sexual function and overall satisfaction following COVID‐19 infection. This observation aligns with the findings of Swedish scholar Rasoal D from March 2023 [16]. The reasons for the decline in sexual function and satisfaction scores post‐infection may include: (1) the psychological impact of COVID‐19, which can persist even after physical symptoms have subsided, and (2) a link between COVID‐19 and erectile dysfunction (ED), which can directly reduce overall satisfaction.

COVID‐19 infection adversely impacts male erectile function across age groups. Our real‐world study of 1098 Chinese males revealed a post‐infection increase in sexual drive contrasting with reduced libido in long‐COVID cohorts. Crucially, we observed significantly increased erectile difficulties, especially among young (18–29) and middle‐aged men (30–44), alongside declines in total sexual function and overall satisfaction, most pronounced in youth. This study establishes COVID‐19 as a significant risk factor for erectile dysfunction, advocating for targeted interventions in younger populations and further mechanistic research. While conflicting evidence exists regarding testicular susceptibility (e.g., discordant ACE2/TMPRSS2 co‐expression reports), histopathological studies confirm SARS‐CoV‐2 presence in testicular tissue via RT‐PCR, IHC, and TEM in specific cohorts, correlating with observed injuries like interstitial edema, vascular changes, and germ cell loss. Direct viral entry is facilitated by ACE2 in Leydig/Sertoli cells and alternative receptors (NRP1/CD147) in the epididymis, alongside viral spike protein binding to epididymal sperm. Indirect mechanisms include systemic inflammation (elevated seminal IL‐6/TNF‐α), febrile insults during spermiogenesis, and endothelial dysfunction leading to erectile dysfunction (eNOS reduction, perivascular viral particles). Furthermore, hypogonadism (linked to Leydig cell damage) and dysregulated reproductive hormones underpin functional impairment. Methodological variations (biopsy timing/technique) contribute to discrepancies, but the aggregate data substantiates both direct infection and immune‐mediated pathophysiology in male reproductive tissues [17, 18].

While conflicting evidence exists regarding testicular susceptibility (e.g., discordant ACE2/TMPRSS2 co‐expression reports), histopathological studies confirm SARS‐CoV‐2 presence in testicular tissue via RT‐PCR, IHC, and TEM in specific cohorts, correlating with observed injuries like interstitial edema, vascular changes, and germ cell loss. Direct viral entry is facilitated by ACE2 in Leydig/Sertoli cells and alternative receptors (NRP1/CD147) in the epididymis, alongside viral spike protein binding to epididymal sperm. Indirect mechanisms include systemic inflammation (elevated seminal IL‐6/TNF‐α), febrile insults during spermiogenesis, and endothelial dysfunction leading to erectile dysfunction (eNOS reduction, perivascular viral particles). Furthermore, hypogonadism (linked to Leydig cell damage) and dysregulated reproductive hormones underpin functional impairment. Methodological variations (biopsy timing/technique) contribute to discrepancies, but the aggregate data substantiate both direct infection and immune‐mediated pathophysiology in male reproductive tissues [19].

This result is particularly pronounced in the youth group, likely due to the demographic characteristics of younger individuals who typically have higher levels of sexual function and activity, making them more sensitive to changes in sexual function and satisfaction.

The impact of the novel coronavirus has been profound on human society, significantly affecting male sexual function, which plays a crucial role in an individual's psychological well‐being, marital relationships, and social stability. Thus, the consequences of impaired male sexual function following COVID‐19 infection warrant significant attention.

The data from this study indicate variations in sexual impulse levels, erectile function, sexual satisfaction, and overall satisfaction before and after COVID‐19 infection. The results demonstrate a close relationship between COVID‐19 infection and the new diagnosis of ED. Alongside in‐depth research, it is essential to conduct educational activities for the public, encourage individuals with related symptoms to manage anxiety and depression, and provide support to enhance male sexual life satisfaction and overall quality of life.

However, this study has certain limitations. It compared the sexual function of individuals infected with COVID‐19 and those who were not infected. However, due to the subjective nature of the questionnaire used to evaluate erectile function, as well as the lack of assessment of erectile function (IIEF‐15) before COVID‐19 infection, there may be discrepancies between the reported results and actual outcomes.

## 5 Conclusion

COVID‐19 infection adversely impacts male erectile function across age groups. Our real‐world study of 1098 Chinese males revealed a post‐infection increase in sexual drive contrasting with reduced libido in long‐COVID cohorts. Crucially, we observed significantly increased erectile difficulties, especially among young (18–29) and middle‐aged men (30–44), alongside declines in total sexual function and overall satisfaction, most pronounced in youth. This study establishes COVID‐19 as a significant risk factor for erectile dysfunction, advocating for targeted interventions in younger populations and further mechanistic research.

## Author Contributions

**Dong Liu:** funding acquisition, project administration, supervision. **Chuhong Chen:** conceptualization, data curation, funding acquisition, methodology, supervision, writing – original draft, writing – review and editing. **Zihan Zhou:** data curation, investigation, writing – review and editing. **Zhecheng Zhang:** data curation, formal analysis, validation. **Qingtong Yi:** data curation, formal analysis, visualization. **Rujian Zhu:** formal analysis, writing – review and editing. **Xujun Xuan:** conceptualization, formal analysis, methodology, writing – review and editing.

## Ethics Statement

The study protocol was approved by the Ethics Committee of Pudong Hospital affiliated with Fudan University, adhering to the principle of voluntary participation.

## Consent

Informed consent to participate was obtained from all of the participants in the study. Researchers provided detailed information about the study to participants before conducting the survey.

## Conflicts of Interest

The authors declare no conflicts of interest.

## Supporting information


**Data S1:** ccr371455‐sup‐0001‐supinfo.pdf

## Data Availability

The data underlying this article will be shared on reasonable request to the corresponding author.
