# Supplementary material for: Real‐World Study on the Impact of Novel Coronavirus Infection on Male Erectile Function
Source: Clin Case Rep. 2025 Nov 23;13(11):e71455. doi: 10.1002/ccr3.71455 (PMC12640784; doi:10.1002/ccr3.71455)
Supplement: Supplementary file 1 — Data S1: ccr371455‐sup‐0001‐supinfo.pdf [file CCR3-13-e71455-s001.pdf]

Questionnaire on male sexual function after COVID-19 infection in the Affiliated Hospital of Fudan University

Revered sir:

Shalom! First thanks for the participation. This questionnaire takes about 5 minutes, thank you for your cooperation!

This questionnaire is filled in anonymously, and the information involved is only for research. Any information you provide will be kept strictly confidential. Please rest assured!

Please note: there are no right or wrong options in the questionnaire, please fill them out according to your actual situation and feelings. Thanks again for your participation!

* **Year of your birth**?

please choose

* Is your marital status?

- ○ Unmarried and no girlfriend (end of the answer)
- ○ Unmarried have a girlfriend, but no sex (end of the answer)
- ○ Unmarried have a girlfriend, have a sex life
- ○ Married
- ○ Divorced
- ○ Remarried
- ○ Widowed

* This question sets up the jump logic

*** How many centimeters is your height?**

please choose

*** How many kilograms (kg) is your weight?**

*** What is your current job?**

please choose

* What is your education level?

- ○ Graduate student or above
- ○ University and Junior College
- ○ High School and Technical Secondary School
- ○ Junior High School and below

* **How much is your monthly income?**
  - ○ Has no income
  - ○ <2000
  - ○ 2000-3999
  - ○ 4000-6999
  - ○ >7000

* **Do you smoke any cigarettes?**
  - ○ Now smoking (smoking more than 1 dose a day for more than 6 months)
  - ○ Occasionally smoking (smoking, but not daily or less than six months)
  - ○ Before smoking, now quit smoking
  - ○ Never smoked

* **Have you been drinking alcohol in the past year?**
  - ○ Has never drunk it
  - ○ Once or less than once per month
  - ○ 2 to 4 times per month
  - ○ 2 to 3 times a week
  - ○ At least 4 times per week

* **Do you usually exercise?**
  - ○ Regular exercise (at least 3 times a week for more than 30 minutes)
  - ○ Occasional exercise (exercise 1~3 times per week)
  - ○ Little exercise (<1 exercise per week)

○ Inactivity (exercise <once per month)

* **What is the city you live for a long time?**

* **Your home address?**

○ town

○ rural area

* **Your COVID-19 vaccination status?**

○ was not inoculated

○ 1 dose (dose)

○ 2 doses (dose)

○ 3 doses (dose)

○ 4 doses (dose)

* What is the type of Novel Coronavirus vaccine you received? [Multiple choice]

□ Inactivated vaccine (Sinovac / Sinopharm)

□ Adenovirus vector vaccine (Concino)

□ Recombinant protein vaccine (Zhifei)

□ Other

Depend on [your COVID-19 vaccine to receive..] Option 2,3,4, and 5

*** Have you ever taken any previous medication to improve your sexual function?**

○ Novel coronavirus was used before infection

○ Novel coronavirus was used after infection

○ It has been used both before infection with and after infection with the novel coronavirus

○ Not infected with the novel coronavirus, once used

○ has never been used

*** What are the drugs you have taken to improve sexual function? [Multiple choice]**

□ Western medicine (such as Viagra, Viagra, Ginger, sildenafil or other tadalafil as the main ingredients)

□ Traditional Chinese medicine or Chinese patent medicine (such as Zuogui pill, Yougui pill, Wuzi Yanzong pill, Liuwei Dihuang pill, Compound Xuanju capsule, etc.)

□ Have never taken any related drug

Depend on [whether you take it before...] Option 1,2,3, and 4

*** In the past way have you confirmed infection with novel coronavirus?**

○ Nucleic acid or antigen test positive

○ others

○ Novel coronavirus (jump to [in the past 4 weeks,..])

* This question sets up the jump logic

*** How many times have you ever been infected with novel coronavirus?**

○ 1

○ 2

○ ≥3

Depend on [what are you always through...] Option 1, and 2

*** Do you have the following symptoms during theCOVID-19 diagnosis? [Multiple choice]**

□ Fever

□ Pharyngalgia

□ Cough, increased breathing rate, and even difficulty

□ nasal plug, runny nose

□ Smell or taste decreased □ conjunctivitis

□ Gastrointestinal symptoms: poor appetite, vomiting, diarrhea, and constipation

□ Systemic symptoms: fatigue, easy fatigue, headache, muscle pain, poor spirit

□ Psychological symptoms: low mood or instability □ others

□ has no performance

Depend on [what are you always through...] Option 1, and 2

*** Do you have a chest imaging examination (chest CT or X-ray) during the novel coronavirus infection diagnosis?**

○ Yes

○ No

Depend on [you have previously been infected with a new...] Option 1,2, and 3

*** Chest imaging during novel coronavirus infection diagnosis Does the examination indicate pneumonia -like changes?**

○ have

○ not have

Depend on the [novel coronavirus sense...] Option 1

*** What types of drugs have you taken during the diagnosis? [Multiple choice]**

□ Antipyretic and analgesic drugs (such as ibuprofen, acetaminophen, Merrill, etc.)

□ Traditional Chinese medicine (such as Qingfei detoxification soup, Lotus Qingwen capsule, Jing preventive agent, Qingfei detoxification granules, Jinhua Qinggan granules, Huoxiang Zhengqi Capsule, etc.)

□ Antiviral drugs (e. g., Pfizer Paxlovid, azfudine, monolavir, ambizumab / roistezumab, COVID-19 immunization...

□ Antibiotics (e. g. cephalosporins, clindamycin, amoxicillin, ofloxacin, etc.)

□ Glucocorticosteroids (e. g. dexamethasone, prednisone, methylprednisone, betamethasone, beclomethasone propionate, prednisolone, hydrogenovine.

□ Other drugs

□ Was not taken

Depend on [what are you always through...] Option 1, and 2

*** Have you now recovered from the novel coronavirus infection?**

○ Yes (has recovered)

○ No (not yet recovered, still symptomatic)

Depend on [what are you always through...] Option 1, and 2

*** When did you recover from the Novel Coronavirus infection? (From diagnosis or onset of symptoms to nucleic acid or antigen conversion, or disappearance of symptoms)**

○ for 1-2 days

○ 3-5 days

○ 5-7 days

○ 8-14 days

○ for 15-30 days

○ More than 30 days

Depend on [what are you always through...] Option 1, and 2

*** How long have you been recovering from novel coronavirus infection?**

○ <4weeks
○ 4-8weeks
○ 8-12 weeks
○ >12 weeks

Depend on [do you currently have...] Option 1

*** Before the coronavirus infection was diagnosed, How often you were sexually impulsive?**

○ Not have
○ Few
○ Occasionally
○ Frequent ○

Depend on [what are you always through...] Option 1, and 2

*** How do you evaluate your disease before the novel coronavirus infection is confirmed Sexual impulse level?**

○ Not have
○ Very low
○ Medium
○ Medium high
○ High

Depend on [what are you always through...] Option 1, and 2

*** How often does a complete or partial genital erection occur when you receive any form of sexual stimulation before the novel coronavirus infection is diagnosed?**

○ Not have

○ Few

○ Occasionally

○ Frequent

*** How often can a Novel Coronavirus infection strongly perform sexual intercourse during your genital erection before the infection is confirmed?**

○ Not have

○ Few

○ Occasionally

○ Frequent

Depend on [what are you always through...] Option 1, and 2

*** How difficult is it for your genital erection before the novel coronavirus infection is confirmed?**

○ Had no erection

○ Very difficult

○ Medium

○ Little difficulty

○ No difficulty

Depend on [what are you always through...] Option 1, and 2

*** How difficult was your genital ejaculation when you were sexually stimulated before your novel coronavirus infection was confirmed?**

○ Asexual stimulation

○ Very difficult

○ Medium

○ Little difficulty

○ No difficulty

Depend on [what are you always through...] Option 1, and 2

*** Before the novel coronavirus infection is confirmed, you think your ejaculation What is the question of quantity?**

○ Did not reach a climax

○ Major issues

○ Secondary

○ A minor question

○ No problem

Depend on [what are you always through...] Option 1, and 2

*** How much problem do you think it is for you before you are diagnosed with reduced sexual impulse?**

○ Big problem ○ medium

○ A minor question

○ Very small problems

○ Is not a problem

Depend on [what are you always through...] Option 1, and 2

*** How much problem do you think it is to induce or maintain erectile function before the novel coronavirus infection is confirmed?**

○ Big problem ○ medium

○ a minor question

○ Very small problems

○ is not a problem

Depend on [what are you always through...] Option 1, and 2

*** How big a problem do you think your ejaculation is before the novel coronavirus infection is diagnosed?**

○ Big problem ○ medium

○ A minor question

○ Very small problems

○ Is not a problem

Depend on [what are you always through...] Option 1, and 2

*** Overall, before the novel coronavirus infection is confirmed, you know How satisfied are you with your sex life?**

○ Very dissatisfied

○ Most people are dissatisfied

○ Insentience

○ Most people are satisfied

- ○ Very satisfied

Depend on [what are you always through...] Option 1, and 2

*** After the novel coronavirus infection recovery, How often you are sexually impulsive?**

- ○ Not have
- ○ Few
- ○ Occasionally
- ○ Frequent

Depend on [what are you always through...] Option 1, and 2

*** How do you evaluate yours after the recovery from the novel coronavirus infection Sexual impulse level?**

- ○ Not have
- ○ Very low
- ○ Medium
- ○ High

Depend on [what are you always through...] Option 1, and 2

*** How often does a complete or partial genital erection occur when you receive any form of sexual stimulation?**

- ○ Not have
- ○ Few
- ○ Occasionally
- ○ Frequent ○

Depend on [what are you always through...] Option 1, and 2

*** After the Novel Coronavirus infection recovers, when your genitals thrive How often can it consistently complete sexual intercourse?**

- ○ Not have
- ○ Few
- ○ Occasionally
- ○ Frequent

Depend on [what are you always through...] Option 1, and 2

*** How difficult is it for your genital erection after your recovery from novel coronavirus infection?**

- ○ Had no erection
- ○ Very difficult
- ○ Medium
- ○ Little difficulty
- ○ No difficulty

Depend on [what are you always through...] Option 1, and 2

*** After the novel coronavirus infection recovery, when you are sexually stimulated How difficult is your genital ejaculation?**

- ○ asexual stimulation
- ○ Very difficult
- ○ medium
- ○ Little difficulty
- ○ No difficulty

Depend on [what are you always through...] Option 1, and 2

*** How much problem do you think your ejaculation is after your recovery from the coronavirus infection?**

○ Has not reached an orgasm
○ big problem
○ secondary
○ Small problems
○ OK

Depend on [what are you always through...] Option 1, and 2

*** After novel coronavirus infection recovery, how much problem do you think reducing sexual impulse is for you?**

○ Big problem ○ medium
○ A minor question
○ Very small problems
○ Is not a problem

Depend on [what are you always through...] Option 1, and 2

*** How much problem do you think it is to induce or maintain erectile function?**

○ Big problem
○ Medium
○ a minor question
○ Very small problem.

- ○ Is not a problem

*** After the recovery from the novel coronavirus infection, you consider your own shot How big is it really a problem?**

- ○ major issues
- ○ secondary
- ○ a minor question
- ○ Very small problems
- ○ is not a problem

Depend on [what are you always through...] Option 1, and 2

*** In general, how are you satisfied with your sexual life after recovering from novel coronavirus infection?**

- ○ was very dissatisfied
- ○ Most people are dissatisfied
- ○ insentience
- ○ is mostly satisfied
- ○ was very satisfied

Depend on [what are you always through...] Option 1, and 2

The following is about your sexual function in the last 4 weeks (or 30 days)

1* How often were you able to get an erection during sexual activity?

- ○ 0 No sexual activity
- ○ 1 Almost never or never
- ○ 2 A few times (less than half the time)
- ○ 3 Sometimes (about half the time)
- ○ 4 Most times (more than half the time)
- ○ 5 Almost always or always

2* When you had erections with sexual stimulation, how often were your erections hard enough for penetration?

- ○ 0 No sexual activity
- ○ 1 Almost never or never
- ○ 2 A few times (less than half the time)
- ○ 3 Sometimes (about half the time)
- ○ 4 Most times (more than half the time)
- ○ 5 Almost always or always

3* When you attempted intercourse, how often were you able to penetrate (enter) your partner?

- ○ 0 Did not attempt intercourse
- ○ 1 Almost never or never
- ○ 2 A few times (less than half the time)
- ○ 3 Sometimes (about half the time)
- ○ 4 Most times (more than half the time)
- ○ 5 Almost always or always

4* During sexual intercourse, how often were you able to maintain your erection after you had penetrated (entered) your partner?

○ 0 Did not attempt intercourse
○ 1 Almost never or never
○ 2 A few times (less than half the time)
○ 3 Sometimes (about half the time)
○ 4 Most times (more than half the time)
○ 5 Almost always or always

5* During sexual intercourse, how difficult was it to maintain your erection to completion of intercourse?

○ 0 Did not attempt intercourse
○ 1 Extremely difficult
○ 2 Very difficult
○ 4 Slightly difficult
○ 5 Not difficult

6* How many times have you attempted sexual intercourse?

○ 0 No attempts
○ 1 One to two attempts
○ 2 Three to four attempts
○ 3 Five to six attempts
○ 4 Seven to ten attempts
○ 5 Eleven or more attempts

7* When you attempted sexual intercourse, how often was it satisfactory for you?

○ 0 Did not attempt intercourse
○ 1 Almost never or never
○ 2 A few times (less than half the time)
○ 3 Sometimes (about half the time)
○ 4 Most times (more than half the time)
○ 5 Almost always or always

8* How much have you enjoyed sexual intercourse?

○ 0 No intercourse
○ 1 No enjoyment at all
○ 2 Not very enjoyable
○ 3 Fairly enjoyable
○ 4 Highly enjoyable
○ 5 Very highly enjoyable

9* When you had sexual stimulation or intercourse, how often did you ejaculate??

○ 0 No sexual stimulation or intercourse
○ 1 Almost never or never
○ 2 A few times (less than half the time)
○ 3 Sometimes (about half the time)
○ 4 Most times (more than half the time)
○ 5 Almost always or always

10* When you had sexual stimulation or intercourse, how often did you have the feeling of orgasm or climax?

- ○ 1 Almost never or never
- ○ 2 A few times (less than half the time)
- ○ 3 Sometimes (about half the time)
- ○ 4 Most times (more than half the time)
- ○ 5 Almost always or always

11* How often have you felt sexual desire?

- ○ 1 Almost never or never
- ○ 2 A few times (less than half the time)
- ○ 3 Sometimes (about half the time)
- ○ 4 Most times (more than half the time)
- ○ 5 Almost always or always

12* How would you rate your level of sexual desire?

- ○ 1 Very low or none at all
- ○ 2 Low
- ○ 3 Moderate
- ○ 4 High
- ○ 5 Very high

13* How satisfied have you been with your overall sex life?

- ○ 1 Very dissatisfied
- ○ 2 Moderately dissatisfie

○ 3 Equally satisfied & dissatisfied

○ 4 Moderately satisfied

○ 5 Very satisfied

14* How satisfied have you been with your sexual relationship with your partner?

○ 1 Very dissatisfied

○ 2 Moderately dissatisfie

○ 3 Equally satisfied & dissatisfied

○ 4 Moderately satisfied

○ 5 Very satisfied

15* How do you rate your confidence that you could get and keep an erection?

○ 1 Very low

○ 2 Low

○ 3 Moderate

○ 4 High

○ 5 Very high

* How much question do you think your ejaculation is in the last 4 weeks subject?

○ Big problem

○ medium

○ a minor question

○ Very small problems

○ is not a problem

* Whether the size of the testis has changed in the last half year, or before and after COVID-19 infection spend?

- ○ There were no significant changes on either side
- ○ Ttestis (one side or side)
- ○ Sticular s of slightly s (one side or side)
- ○ Increticular s enlargement (one or both)
